# Extensive Immunological and Inflammatory Perturbation Underpins the Respiratory Sequelae of Postacute COVID-19

**DOI:** 10.1093/ofid/ofag050

**Published:** 2026-02-05

**Authors:** Gang Yang, Jinpeng Cao, Shidong Deng, Yingjiao Xia, Jun Zhao, Jian Qin, Xiaoyun Yang, Min Sun, Daxiang Chen, Ping Sun, Yunhui Zhang, Zheng Deng, Deyi Huang, Shiqin Jin, Tianyang Fu, Nanshan Zhong, Zhongfang Wang

**Affiliations:** State Key Laboratory of Respiratory Disease & National Clinical Research Center for Respiratory Disease, Guangzhou Institute of Respiratory Health, The First Affiliated Hospital of Guangzhou Medical University, Guangzhou Medical University, Guangzhou, China; Guangzhou National Laboratory, Bioland, Guangzhou, Guangdong, China; The Affiliated Hospital of Kunming University of Science and Technology. Department of Respiratory and Critical Care Medicine, The First People's Hospital of Yunnan Province, Kunming, Yunnan, China; Yunnan Baiyao Group Co., Ltd., Central Research Institute, Kunming, Yunnan, China; State Key Laboratory of Respiratory Disease & National Clinical Research Center for Respiratory Disease, Guangzhou Institute of Respiratory Health, The First Affiliated Hospital of Guangzhou Medical University, Guangzhou Medical University, Guangzhou, China; Guangzhou National Laboratory, Bioland, Guangzhou, Guangdong, China; State Key Laboratory of Respiratory Disease & National Clinical Research Center for Respiratory Disease, Guangzhou Institute of Respiratory Health, The First Affiliated Hospital of Guangzhou Medical University, Guangzhou Medical University, Guangzhou, China; Guangzhou National Laboratory, Bioland, Guangzhou, Guangdong, China; The Affiliated Hospital of Kunming University of Science and Technology. Department of Respiratory and Critical Care Medicine, The First People's Hospital of Yunnan Province, Kunming, Yunnan, China; The Affiliated Hospital of Kunming University of Science and Technology. Department of Respiratory and Critical Care Medicine, The First People's Hospital of Yunnan Province, Kunming, Yunnan, China; The Affiliated Hospital of Kunming University of Science and Technology. Department of Respiratory and Critical Care Medicine, The First People's Hospital of Yunnan Province, Kunming, Yunnan, China; State Key Laboratory of Respiratory Disease & National Clinical Research Center for Respiratory Disease, Guangzhou Institute of Respiratory Health, The First Affiliated Hospital of Guangzhou Medical University, Guangzhou Medical University, Guangzhou, China; Guangzhou National Laboratory, Bioland, Guangzhou, Guangdong, China; Yunnan Baiyao Group Co., Ltd., Central Research Institute, Kunming, Yunnan, China; State Key Laboratory of Respiratory Disease & National Clinical Research Center for Respiratory Disease, Guangzhou Institute of Respiratory Health, The First Affiliated Hospital of Guangzhou Medical University, Guangzhou Medical University, Guangzhou, China; Guangzhou National Laboratory, Bioland, Guangzhou, Guangdong, China; The Affiliated Hospital of Kunming University of Science and Technology. Department of Respiratory and Critical Care Medicine, The First People's Hospital of Yunnan Province, Kunming, Yunnan, China; The Affiliated Hospital of Kunming University of Science and Technology. Department of Respiratory and Critical Care Medicine, The First People's Hospital of Yunnan Province, Kunming, Yunnan, China; The Affiliated Hospital of Kunming University of Science and Technology. Department of Respiratory and Critical Care Medicine, The First People's Hospital of Yunnan Province, Kunming, Yunnan, China; State Key Laboratory of Respiratory Disease & National Clinical Research Center for Respiratory Disease, Guangzhou Institute of Respiratory Health, The First Affiliated Hospital of Guangzhou Medical University, Guangzhou Medical University, Guangzhou, China; Guangzhou National Laboratory, Bioland, Guangzhou, Guangdong, China; State Key Laboratory of Respiratory Disease & National Clinical Research Center for Respiratory Disease, Guangzhou Institute of Respiratory Health, The First Affiliated Hospital of Guangzhou Medical University, Guangzhou Medical University, Guangzhou, China; Guangzhou National Laboratory, Bioland, Guangzhou, Guangdong, China; State Key Laboratory of Respiratory Disease & National Clinical Research Center for Respiratory Disease, Guangzhou Institute of Respiratory Health, The First Affiliated Hospital of Guangzhou Medical University, Guangzhou Medical University, Guangzhou, China; Guangzhou National Laboratory, Bioland, Guangzhou, Guangdong, China; State Key Laboratory of Respiratory Disease & National Clinical Research Center for Respiratory Disease, Guangzhou Institute of Respiratory Health, The First Affiliated Hospital of Guangzhou Medical University, Guangzhou Medical University, Guangzhou, China; Guangzhou National Laboratory, Bioland, Guangzhou, Guangdong, China; The Affiliated Hospital of Kunming University of Science and Technology. Department of Respiratory and Critical Care Medicine, The First People's Hospital of Yunnan Province, Kunming, Yunnan, China; State Key Laboratory of Respiratory Disease & National Clinical Research Center for Respiratory Disease, Guangzhou Institute of Respiratory Health, The First Affiliated Hospital of Guangzhou Medical University, Guangzhou Medical University, Guangzhou, China; Guangzhou National Laboratory, Bioland, Guangzhou, Guangdong, China

**Keywords:** antibody, complement, inflammation, respiratory sequelae of post-acute COVID-19, T lymphocyte

## Abstract

Postacute sequelae of COVID-19 (PASC), a multisystem disorder with prevalent respiratory manifestations, affecting millions of individuals worldwide, yet the organ-/system-specific PASC pathogenesis and targeted interventions remain largely undefined. In this longitudinal cohort study of individuals followed up at 4 (n = 57) and 7 months (n = 54) after the Omicron BA.5 outbreak in China, we comprehensively analyzed physician-administered PASC symptom assessments, clinical respiratory evaluations (pulmonary function and chest computed tomography), immunological response profiles, and inflammatory markers. Our findings demonstrated that patients with respiratory system–specific PASC (R-PASC) endure long-term pulmonary function impairment (restrictive ventilation and diffusion dysfunction), sustained severe residual lung lesions (predominant fibrosis), and chronic systemic inflammatory responses. Patients with R-PASC exhibited enhanced SARS-CoV-2–specific T-cell responses, whereas in the control group, moderate-magnitude and polyfunctional virus-specific T-cell response correlated with improved lung function and alleviated inflammation. Sustained neutralizing antibody titers were also observed in patients with R-PASC, whereas humoral responses showed minimal association with disease pathophysiology. Moreover, prolonged activation of complement classical and alternative pathway in patients with R-PASC is associated with worsening respiratory parameters, whereas mannose-binding lectin within the lectin pathway exhibits protective correlations with pulmonary tissue function preservation. Overall, our study delineates the extensively perturbed immune–inflammation–organ dysfunction in patients with R-PASC, thereby providing valuable insights into the pathogenesis of this condition and highlighting potential targets for therapeutic intervention.

Following acute infection with SARS-CoV-2, a substantial proportion of patients report persistent pulmonary and multi-system symptoms, termed postacute sequelae of COVID-19 (PASC) or Long COVID. Currently, the estimated global cumulative incidence of PASC is substantial [1–5], with respiratory manifestations constituting a predominant cluster of symptoms (R-PASC), including chronic cough, persistent breathlessness, and dyspnea, lasting months after initial infection, representing a major public health issue [6–8].

In the pathogenic hypotheses of PASC, the persistent residual presence of SARS-CoV-2 is currently recognized as a pivotal upstream triggering factor [4, 9, 10]. Evidence suggests that viral components, including RNA and antigens, can reside in tissue reservoirs for months, potentially driving downstream immunological and inflammatory dysregulation [9]. However, the immune landscape of PASC is characterized by significant heterogeneity across studies. For instance, investigations of virus-specific T cells have yielded inconsistent results across different clinical cohorts [11–13], highlighting the inherent complexity of PASC and the limitations of studying it as a single entity from broadly defined PASC populations. Consequently, this heterogeneity strongly suggests that focusing on specific organ-/system-defined PASC subtypes, particularly the prevalent R-PASC, and employing more uniform case inclusion criteria [14], may provide a more accurate understanding of its pathogenesis and facilitate the development of targeted interventions.

To address these gaps, we established a longitudinal cohort during the Omicron BA.5 wave in China, tracking primarily on hospitalized survivors for 7 months postdischarge. Physician-administered PASC symptom assessments, respiratory function, and pulmonary imaging were evaluated at multiple time points; long-term immunological effectors include SARS-CoV-2–specific T-cell responses, antiviral antibodies, and complement components were assessed; inflammatory cytokines and chemokines in plasma, along with acute viral loads in the upper respiratory tract, were also quantified. By integrating these multidimensional data, we aimed to systematically dissect the pathophysiological mechanisms of R-PASC and to elucidate the complex interrelation among various immune factors in driving disease progression.

## RESULT

### Cohort Characteristic

**Figure 1.**
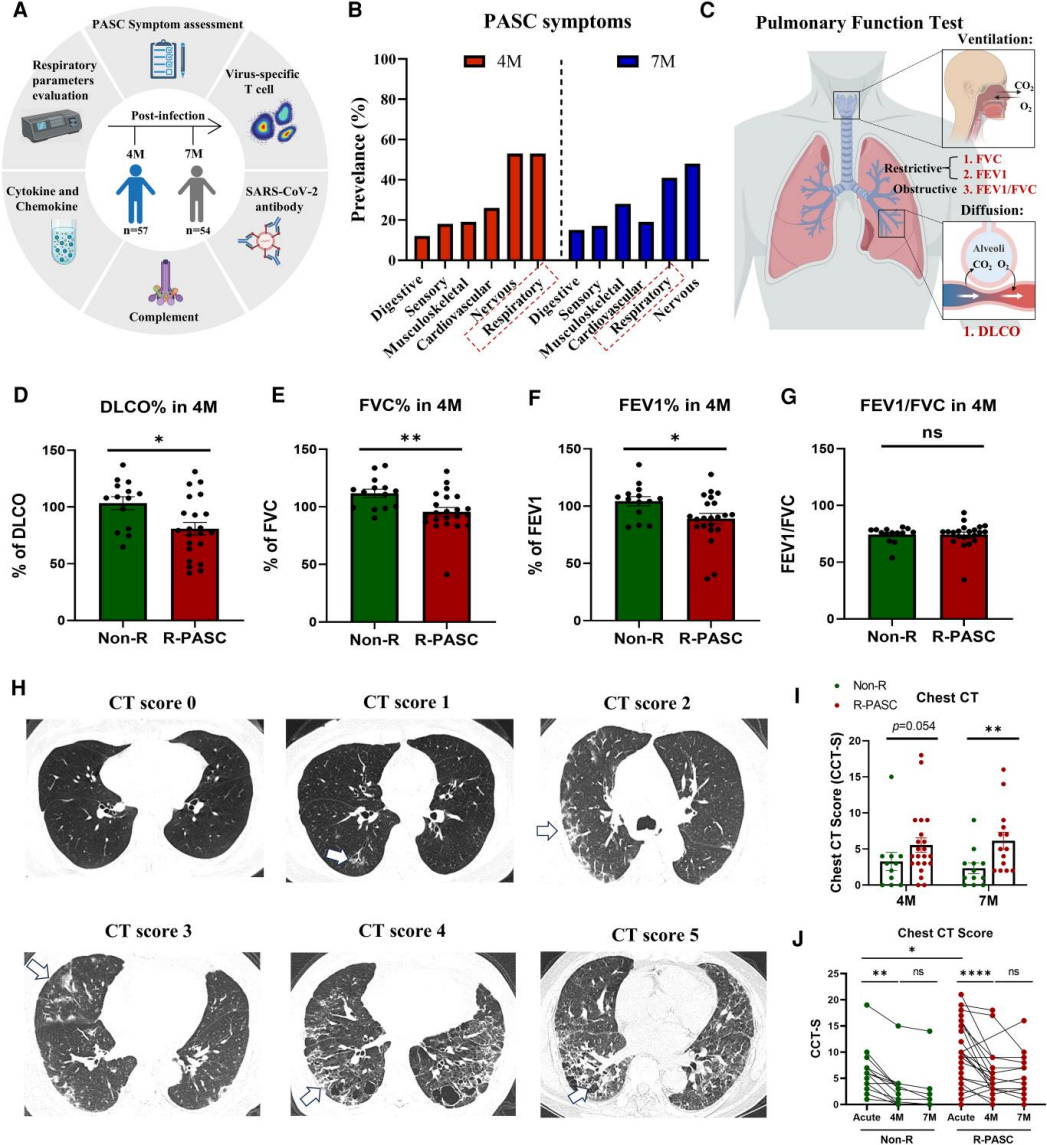
Study cohort and respiratory parameter profiling. *A*, Schematic diagram of the study cohort and experimental design. *B*, Prevalence of PASC-affected organ/systems at 4 M (left) and 7 M (right) postinfection. *C*, Schematic diagram of pulmonary function test and parameters, including ventilation (FVC%, FEV1%, FEV1/FVC ratio) and diffusion capacity (DLCO%). *D–G*, Comparative analysis of pulmonary function parameters between the R-PASC group and non-R group. *H*, Representative chest CT images with semiquantitative assessment of lung lesion severity using the CT scoring system (score scale from 0 to 5). *I*, Comparation of the chest CT severity scores between R-PASC and non-R groups at 4 and 7 M. *J*, Temporal trajectories of lung lesion severity within both groups. Each dot represents 1 donor. Comparisons for paired samples were performed using the Wilcoxon matched-pairs signed rank test. Comparisons between groups were performed using Mann-Whitney tests. *P < .05, **P < .01, ***P < .001. CT, computed tomography; FVC, forced vital capacity; FEV1, first second of forced expiration; M, months; PASC, postacute sequelae of COVID-19; R-PASC, respiratory system–specific PASC.

We established a longitudinal research cohort during the Omicron BA.5 outbreak in China in December 2022 [15], conducting 2 follow-up visits at 4 months (4 M, n = 57) and 7 months (7 M, n = 54) postacute infection (Figure 1*A* and Table 1). At each follow-up, systematic PASC symptom assessments were administered by the respiratory physician, and respiratory parameter evaluations were conducted including pulmonary function tests and chest computed tomography (CT) scan. Among 12 PASC symptoms evaluated, the highest incidence rates were observed for dyspnea (46% at 4 M and 39% at 7 M), fatigue (40% at 4 M and 46% at 7 M), impaired cognitive function (26% at 4 M and 41% at 7 M), and chronic cough (22% at 4 M and 20% at 7 M) (Table 1), which are consistent with the symptom incidence rates reported in previous PASC studies [6, 16]. Further categorizing the symptom clusters into organ/systems, the data indicated that PASC predominantly affects the respiratory system (53% at 4 M and 41% at 7 M) and the nervous system (53% at 4 M and 46% at 7 M) (Figure 1*B* and Table 1). However, during the timeframe from 4 to 7 M, most rates of PASC organ involvements or symptoms remained persistent without significant relief (Table 1). These data suggest that the potential pathogenic factors leading to PASC may persist, or their residual effects may last for an extended period.

**Table 1.** PASC Symptom Characteristics of Cohort Participants

	4 M	7 M	*P* Value
Participants, no.	57	54	
Age, no. (range)	57 (25–87)	56 (25–87)	/
Sex, no. (%)			
Male	34 (60)	33 (61)	/
Female	23 (40)	21 (39)	/
Follow-up time from symptom onset (range)	116 (89–131)	221 (205–234)	/
PASC symptoms, no. (%)			
Chronic cough	13 (22)	11 (20)	.755
Dyspnea	26 (46)	21 (39)	.474
Palpitation	11 (19)	10 (19)	.917
Chest pain	5 (9)	0 (0)	.**026**
Cognitive impairment	15 (26)	22 (41)	.107
Fatigue	23 (40)	25 (46)	.527
Headache	2 (3)	7 (13)	.068
Insomnia	11 (19)	14 (26)	.403
Nausea and anorexia	7 (12)	8 (15)	.696
Hyposmia and hypogeusia	10 (18)	9 (17)	.902
Myalgia	8 (14)	13 (24)	.177
Arthralgia	5 (9)	8 (15)	.322
PASC system involvement, no. (%)			
Respiratory system	30 (53)	22 (41)	.210
Cardiovascular system	15 (26)	10 (19)	.326
Nervous system	30 (53)	25 (46)	.193
Digestive system	7 (12)	8 (15)	.696
Sensory system	10 (18)	9 (17)	.902
Musculoskeletal system	11 (19)	15 (28)	.292

M, month; PASC, postacute sequelae of COVID-19.

### Respiratory Pathophysiological Parameter Characterization

The respiratory system, as the primary site of SARS-CoV-2 invasion, is also a major system affected by PASC. Therefore, we focused on R-PASC and divided the cohort into R-PASC group (n = 30 at 4 M, n = 22 at 7 M) and non-R group (individuals without respiratory sequelae symptoms; n = 27 at 4 M, n = 32 at 7 M) based on the physician-administered PASC symptomatology (Table 1). Then, a subset of hospitalized COVID-19 survivors (n = 39) underwent pulmonary function tests at 4 months (Figure 1*C*). The results revealed impairments in multiple pulmonary function parameters among patients with R-PASC, who exhibited significantly lower diffusion capacity (DLCO%) and restrictive ventilation parameters (forced vital capacity [FVC%] and first second of forced expiration [FEV1%]) compared to the non-R group (*P* < .05, Figure 1*D*–1*F*), whereas no difference was observed in the obstructive ventilation parameters FEV1/FVC (*P* = .9108, Figure 1*G*). Paired data also suggest that the lower pulmonary function in patients with R-PASC was already present during the acute infection phase and remained stable from the acute phase to 4 M for both groups (Supplementary Figure 1*A* and 1*B*).

Persistent lung imaging abnormalities in COVID-19 survivors have been reported in multiple studies [17, 18]. We then employed the Chest CT Score [9, 19] to assess the relationship between pulmonary lesions and R-PASC (Figure 1*H*). The data indicate that patients with R-PASC exhibit prolonged and more severe pulmonary lesions compared to non-R individuals, persisting at least 7 M postinfection (*P* = .054 at 4 M and *P* = .008 at 7 M, Figure 1*I*). Longitudinal imaging investigations revealed that, although both groups exhibited significant resolution of pulmonary lesions from the acute phase to 4 M, the lesion area remained stable with no significant change for both groups from 4 to 7 M (Figure 1*J*), whereas lesion types transitioned from ground-glass opacity and consolidation during the acute infection to reticulation (pulmonary fibrosis) or complete normal during convalescence (Supplementary Figure 1*C*).

Notably, regression model analysis demonstrated that symptomatology-based R-PASC grouping was significantly associated with pulmonary function impairment and CT-detectable lung abnormalities, indicating that these symptoms are not entirely subjective but reflect multiple objective physiological dysfunctions (Supplementary Table 1).

### Persistent Inflammation and Acute High Viral Loads are Important Pathogenic Factors in R-PASC

**Figure 2.**
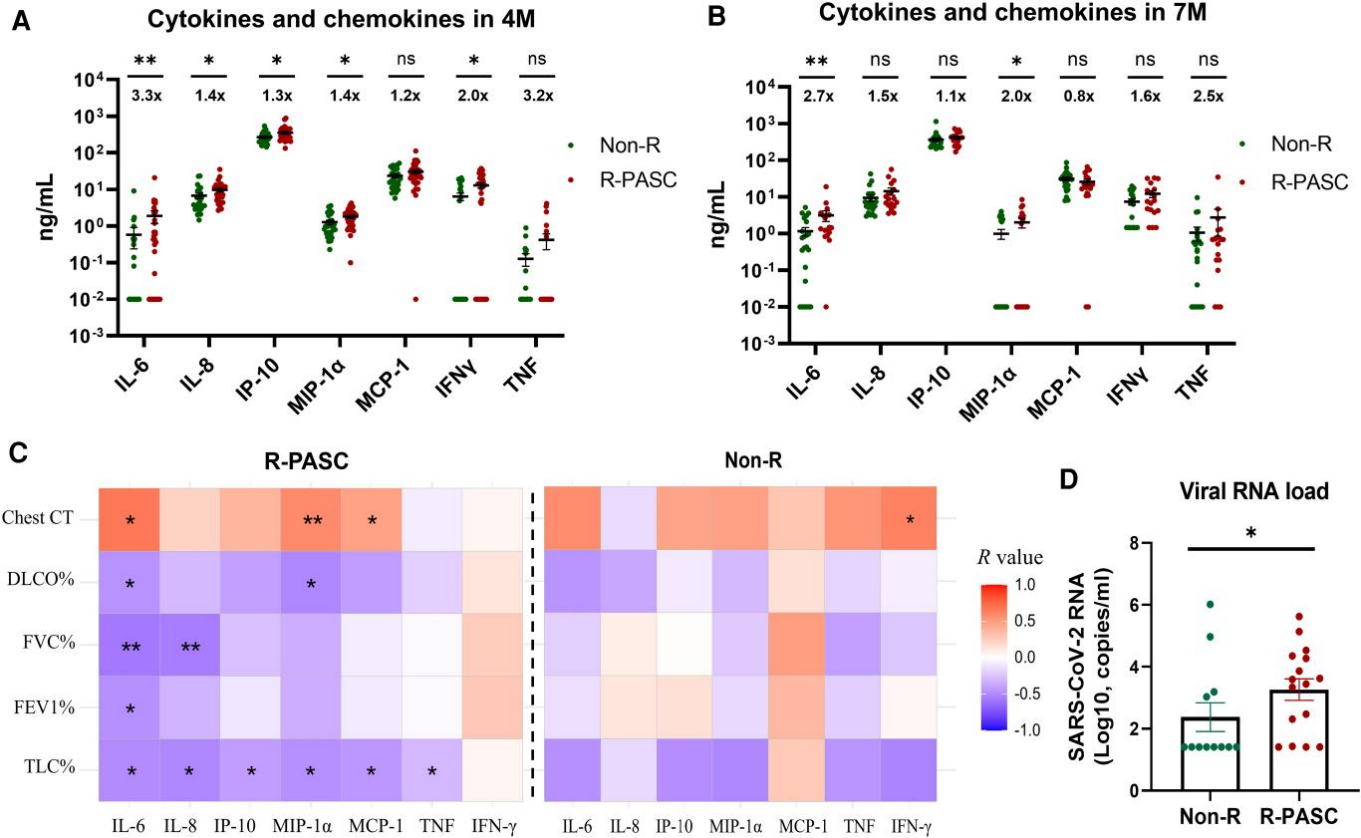
Inflammatory response and acute-phase viral load assessment. *A*, *B*, Longitudinal comparisons of inflammatory cytokine and chemokine levels between the R-PASC and non-R groups at 4 and 7 M are presented, with the fold differences between groups annotated above the scatter plots, using the non-R group values as the baseline. *C*, Correlation heatmap showing the Spearman correlations of inflammatory mediators and respiratory parameters (chest CT scores, FVC%, DLCO%) for the R-PASC group (left) and non-R group (right). *D*, Comparation of the SARS-CoV-2 viral loads in the upper respiratory tract during early infection (<20 days after symptom onset) within each group. Each dot represents 1 donor. Comparisons between groups were performed using Mann-Whitney tests. *P < .05, **P < .01, ***P < .001. CT, computed tomography; DLCO, lower diffusion capacity; FVC, forced vital capacity; FEV1, first second of forced expiration; M, months; PASC, postacute sequelae of COVID-19; R-PASC, respiratory system–specific PASC.

To investigate the potential association between R-PASC and inflammatory responses, we conducted longitudinal profiling of 7 inflammatory mediators, while controlling for reinfection confounders through paired XBB-specific neutralization assays [15]. The results revealed that the R-PASC group exhibited significantly elevated levels of interleukin (IL)-6, IL-8, interferon gamma-induced protein (IP)-10, macrophage inflammatory protein (MIP)-1α, and interferon γ (IFNγ) compared to the non-R group at 4 M postacute infection (*P* < .05, Figure 2*A*). The inflammatory responses persist over the long term, with significantly higher IL-6 and MIP-1α still observed at 7 M in patients with R-PASC (*P* < .05), although overall difference levels between the 2 groups have diminished to some extent (Figure 2*B*). Furthermore, abnormal inflammatory responses in the R-PASC group were significantly correlated with respiratory parameters (Figure 2*C*, left panel), with IL-6, MIP-1α, and monocyte chemoattractant protein (MCP)-1 positively correlating with more severe pulmonary lesions (Chest CT scores), and IL-6, IL-8, IP-10, MIP-1α, and MCP-1 significant negative correlating with various lung function parameters, whereas this effect was scarcely present in the non-R group (Figure 2*C*, right panel). Moreover, given that the SARS-CoV-2 virus itself may be a trigger for PASC, we retrospectively assessed the acute upper respiratory viral load in cohort patients (<20 days postsymptom onset) and found that individuals who later developed R-PASC exhibited significantly higher SARS-CoV-2 RNA loads (Figure 2*D*), suggesting that they experienced a higher dose of antigen exposure.

### Virus-specific T Cells With Multifunctionality and Appropriate Response Magnitude Correlate With Improved Lung Function

**Figure 3.**
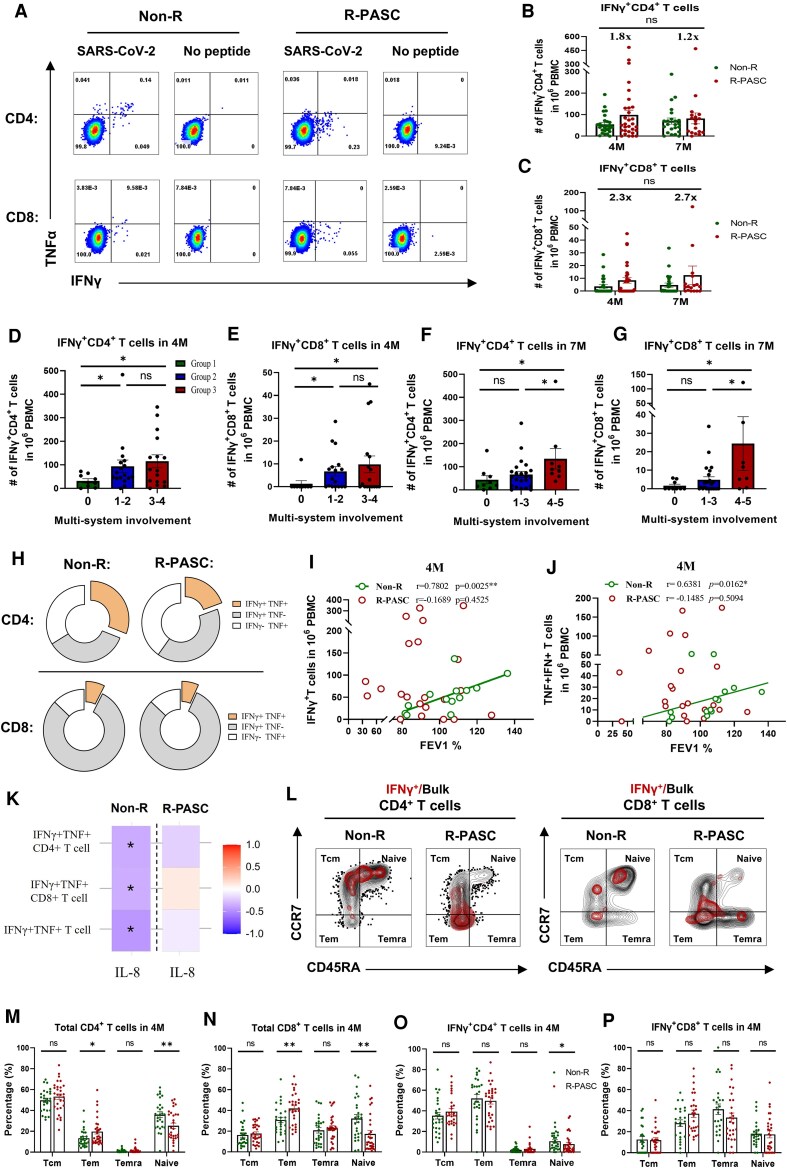
Virus-specific T-cell memory responses and functional correlations. *A*, Representative flow cytometry plots of SARS-CoV-2–specific IFNγ^+^ CD4^+^ and CD8^+^ T-cell responses. *B*, *C*, Comparation of virus-specific CD4^+^ (*B*) and CD8^+^ (*C*) T-cell magnitude between the R-PASC group and non-R group. *D*-*G*, Comparation of virus-specific T-cell magnitude within individuals of different number of PASC organ/system involvement at 4 and 7 M. *H*, Polyfunctionality of virus-specific CD4^+^ and CD8^+^ T cells in R-PASC versus non-R groups. IFNγ^+^TNFα^+^ T-cell subsets highlighted in orange. *I*, *J*, Correlation scatter plots showing the Spearman correlations between total (*I*) and polyfunctional (*J*) virus-specific T cells with pulmonary function FEV1%. *K*, Correlation heatmap showing the Spearman correlations between virus-specific T-cell number and plasma inflammatory mediator IL-8 levels across both groups. *L*, Representative stacked flow cytometry plots showing memory phenotypic distribution of total/bulk (black) versus virus-specific (red) CD4^+^ and CD8^+^ T cells subsets. *M–P*, Comparative analysis of phenotypes frequencies of total/bulk (*O*, *P*) and virus-specific (*M*, *N*) memory T-cell subsets across both groups at 4 M. Each dot represents one donor. Comparisons between groups were performed using Mann-Whitney tests. *P < .05, **P < .01, ***P < .001. FVC, forced vital capacity; FEV1, first second of forced expiration; M, months; PASC, postacute sequelae of COVID-19; R-PASC, respiratory system–specific PASC.

To profile the SARS-CoV-2–specific T-cell response related to PASC, particularly R-PASC, we subsequently measured the number of virus-specific (IFNγ^+^) T cells in longitudinal peripheral blood mononuclear cells (PBMCs) samples (Figure 3*A*). The results showed that virus-specific CD4^+^ and CD8^+^ T cells in patients with R-PASC were trendily higher (1.2-1.8-fold in CD4; 2.3-2.7-fold in CD8) than those in the non-R group at both 4 and 7 M (Figure 2*B* and 2*C*). Furthermore, we observed that the numbers of IFNγ^+^ CD4^+^ and CD8^+^ T cells increased with the number of organ systems affected by PASC (Figure 3*D*–3*G*; grouping details are provided in the Methods). Counts in group 3 (most extensive involvement) were significantly higher than in group 1 (unaffected) at both 4 and 7 M (*P* < .05) and were elevated compared to group 2 (fewer affected systems) with a trend at 4 M and became significant at 7 M (*P* < .05).

Multifunctional virus-specific T cells have been reported to mediate the early clearance of SARS-CoV-2 and be associated with mild infections [20]. Our data suggest that, compared to patients with R-PASC, the virus-specific T-cell population in non-R individuals has greater multifunctionality (IFNγ^+^TNF^+^), particularly within the CD4^+^ T cell subsets (*P* = .0091, Figure 3*H*). More importantly, total and multifunctional virus-specific T-cell responses, especially CD4^+^ subsets, were significantly positively correlated with improved pulmonary function parameters FEV1% and FVC% in the non-R group rather than the R-PASC group (Figure 3*I* and 3*J*, and Supplementary Figure 2*A*–2*D*), suggesting a potential contribution to the protection for lung ventilatory function. Multifunctional virus-specific T cells in non-R are also significantly negatively correlated with the inflammatory cytokine IL-8 (Figure 3*K*). No correlation was observed between virus-specific T cells and the pulmonary lesions in either group at 4 and 7 M (Supplementary Figure 2*E* and 2*F*). In addition, using 2 memory markers (CD45RA and CCR7, Figure 3*L*), we also assessed the memory phenotypes in both groups, finding that patients with R-PASC exhibited significantly lower naïve T cells and higher effector memory T cells in both total and virus-specific T-cell subsets compared to the non-R group (Figure 3*M*–3*P*).

### Sustained Antibody Responses Show Minimal Correlation With R-PASC Pathophysiology

**Figure 4.**
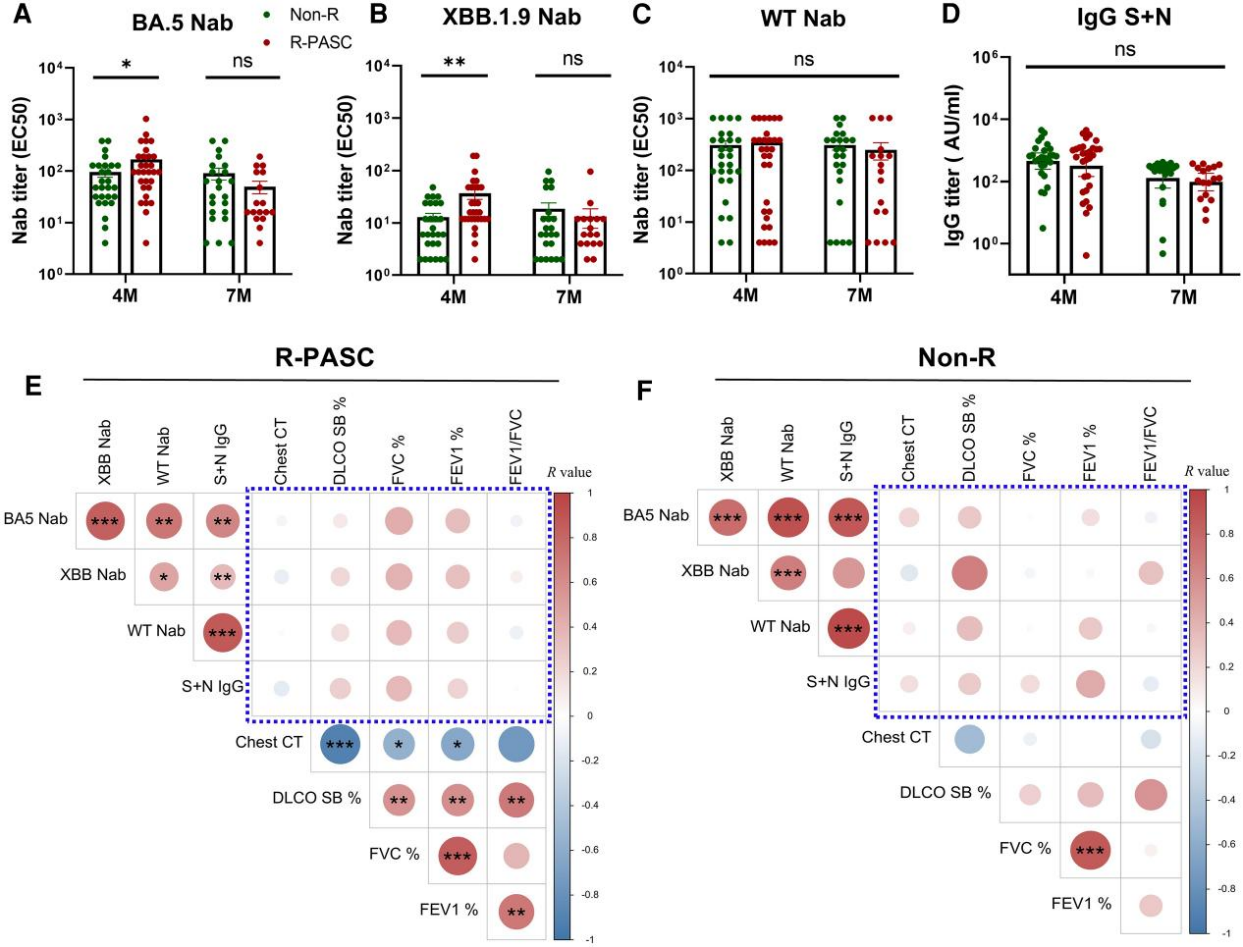
Antivirus antibody responses and pathophysiological correlations. *A–D*, Comparative analysis of longitudinal antibody responses between R-PASC group and non-R group at 4 and 7 M postinfection, including neutralizing antibodies (BA.5 Nab, XBB.1.9 Nab and wild-type Nab) and anti-S + N IgG titers. *E*, *F*, Correlation matrices showing the Spearman correlations between antibody titers and R-PASC-relevant pathophysiological parameters (pulmonary function and CT scores) within R-PASC group (*E*) and non-R group (*F*). Each dot represents 1 donor. Comparisons between groups were performed using Mann-Whitney tests. *P < .05, **P < .01, ***P < .001. CT, computed tomography; M, months; PASC, postacute sequelae of COVID-19; R-PASC, respiratory system–specific PASC.

**Figure 5.**
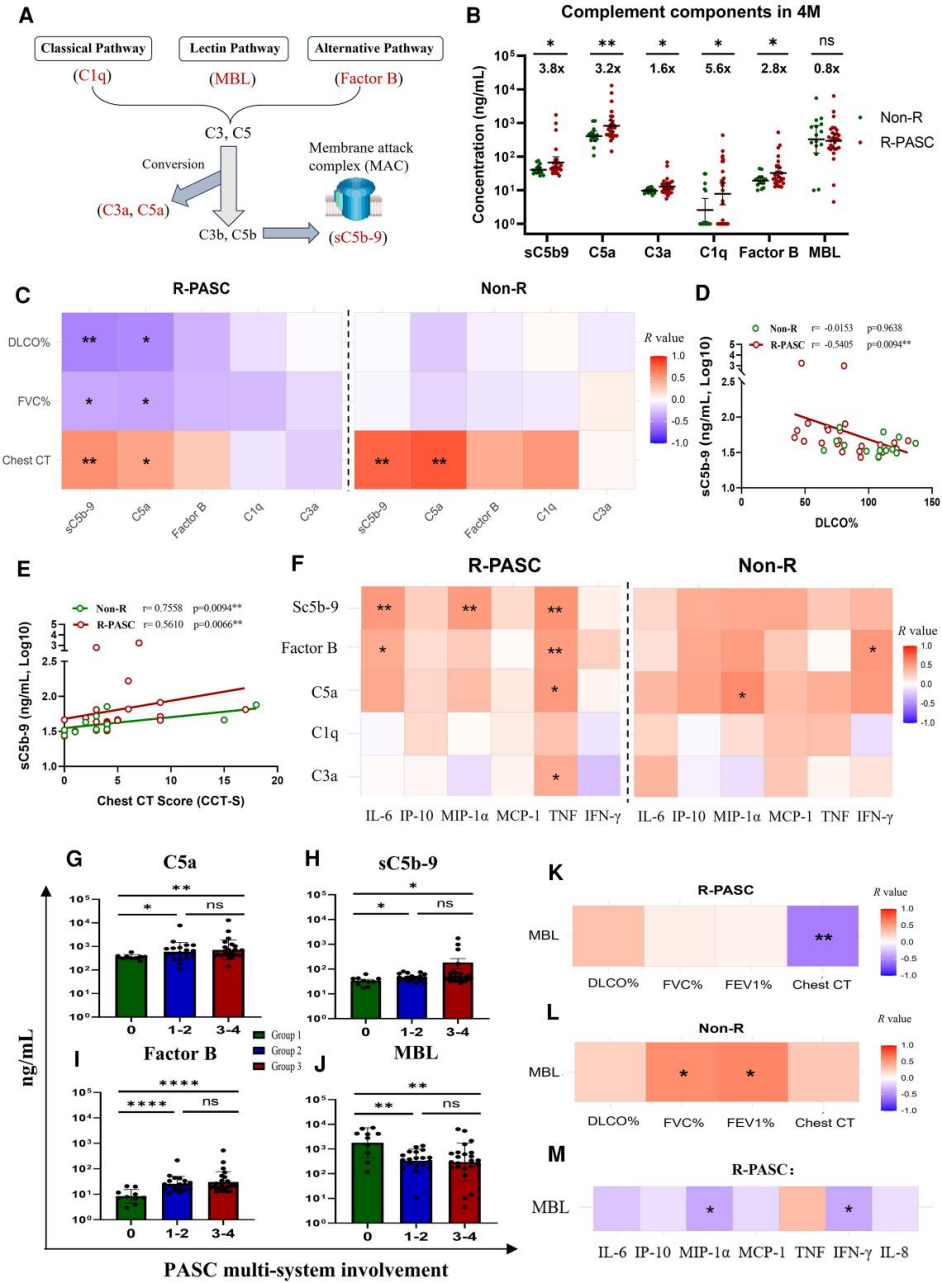
Complement system assessment and pathophysiological correlations. *A*, Schematic diagram of complement activation pathways (classical, lectin and alternative pathways), with red-highlighted nodes indicating quantified complement biomarkers in this study. *B*, Comparison of complement proteins (Sc5b-9, C3a, C5a, C1q, MBL, and Factor B) levels between patients with R-PASC and non-R controls are presented, with the fold differences between groups annotated above the scatter plots, using the non-R group values as the baseline. *C–E*, Correlation analysis showing the Spearman correlations between complement activation markers and respiratory parameters (lung function and chest CT scores) within 2 groups. *F*, Correlation heatmap showing the Spearman correlations between complement protein and inflammatory mediators within both group (R-PASC in left panel and non-R in right panel). *G–J*, Comparison of complement protein levels in individuals with different number of PASC-affected organ/systems. *K–M*, Correlation heatmap showing the Spearman correlations of MBL in lectin pathway with lung function, chest CT scores, and inflammatory mediators. Each dot represents 1 donor. Comparisons between groups were performed using Mann-Whitney tests. *P < .05, **P < .01, ***P < .001. CT, computed tomography; PASC, postacute sequelae of COVID-19.

We subsequently investigated the role of antiviral antibodies in PASC and found that the titer levels of the BA.5 neutralizing antibody (Nab) and cross-reactive XBB.1.9 Nab were significantly higher in the R-PASC group compared to the non-R group at 4 M (Figure 4*A* and 4*B*). However, for wild-type Nab (Figure 4*C*), which is commonly regarded as a baseline type associated with previous vaccination, as well as the binding antibodies S + N IgG (Figure 4*D*), no significant differences were observed between the 2 groups at 4 M. The findings indicate enhanced variant-specific humoral responses in early-stage R-PASC, likely related to their ongoing immune reactions, but not across all antibody types. Notably, for the long-term time point of 7 M, no differences were found in any antibody type between the R-PASC and non-R groups (Figure 4*A*–4*D*), nor between the groups classified by the number of PASC-affected systems (Supplementary Figure 3*A*–3*C*). Furthermore, we analyzed the correlation between antiviral antibody responses and respiratory parameters or inflammatory responses, finding that none of the antibody components showed significant correlations with lung function parameters or the pulmonary lesions in both groups (Figure 4*E* and 5*F* blue frame, Supplementary Figure 3*D*–3*H*), which further suggests that SARS-CoV-2 antibodies may play a minimal or nondirect role in the R-PASC pathophysiology.

### Prolonged Complement Dysregulation in R-PASC

The complement system is another important immunological factor in the PASC pathophysiology. We assessed multiple complement proteins in cohort serum (Figure 5*A*) and found that patients with R-PASC exhibited a wide and excessive complement activation compared to non-R individuals at 4 M (Figure 5*B*), with significantly elevated (*P* < .05) markers in terminal pathway (sC5b-9, C5a, C3a), the classical pathway (C1q), and the alternative pathway (Factor B), whereas the lectin pathway (mannose-binding lectin [MBL]) showed no significant differences between the 2 groups (*P* = .462). Correlation analyses between the complement profiles and respiratory parameters revealed excessively activated sC5b-9 and C5a in the R-PASC group negatively correlated with pulmonary function (DLCO% and FVC%) (*P* < .05), whereas no correlation of complement responses and lung function impairment was found in the non-R group (Figure 5*C* and 5*D*). For lung tissue lesions, both groups exhibited a positive correlation between sC5b-9, C5a, and chest CT scores (*P* < .05, Figure 5*C* and 5*E*), which is consistent with previous studies that observed terminal complement components causing lung injury in hospitalized patients with COVID-19 [21]. Concurrently, the complement components in both groups were positively associated with the inflammatory responses to some extent (Figure 5*F*), suggesting a potential mechanism underlying their injury effects.

Furthermore, as predicted, we observed that as the number of PASC-affected organ systems increased, the activation degree of the complement system correspondingly escalated (Figure 5*G*–5*I* and Supplementary Figure 3*G* and 3*H*), with levels of broad markers in group 3 and group 2 significantly higher than those in group 1 (*P* < .05). Interestingly, the key component of the lectin pathway, MBL, exhibits a distinct role: its level is highest in group 1, which has the least number of affected organ systems (Figure 5*J*), and shows positive correlation with improvements in pulmonary function, lung tissue lesions (Figure 5*K* and 5*L*), and demonstrates negative correlation with inflammatory response in R-PASC (Figure 5*M*). Overall, our data suggest that prolonged activation of the complement response, particularly via the terminal, classical, and alternative pathways, may contribute to the progression of PASC, especially R-PASC, while the lectin pathway may play a protective role.

### Acute-phase Features in Patients Developing Subsequent R-PASC

Previous evidence indicates that severe SARS-CoV-2 infection during the acute phase is associated with the elevated risk of subsequent PASC among hospitalized patients [2, 6, 22]. Therefore, we further analyzed the clinical data from the acute infection phase of individuals with the subsequent respiratory subtype of PASC, and found that compared to the non-R group, patients with R-PASC had higher rates of hospitalization, greater disease severity, and increased requirements for oxygen supplementation (Supplementary Table 2). In addition, a retrospective analysis of clinical laboratory data from the acute infection phase was also performed, regarding parameters including induced sputum cytology, fractional exhaled nitric oxide, complete blood count, blood biochemistry, coagulation profile, and autoantibody profile. Most measured parameters were comparable between the 2 groups (Supplementary Table 2), with the exception of significantly higher lactate dehydrogenase levels in the R-PASC cohort—a marker associated with persistent airway injury following SARS-CoV-2 infection [23]. Collectively, these findings suggest that the acute-phase disease status and related laboratory parameters may serve both biological and predictive roles in the pathophysiological process of R-PASC.

## DISCUSSION

**Figure 6.**
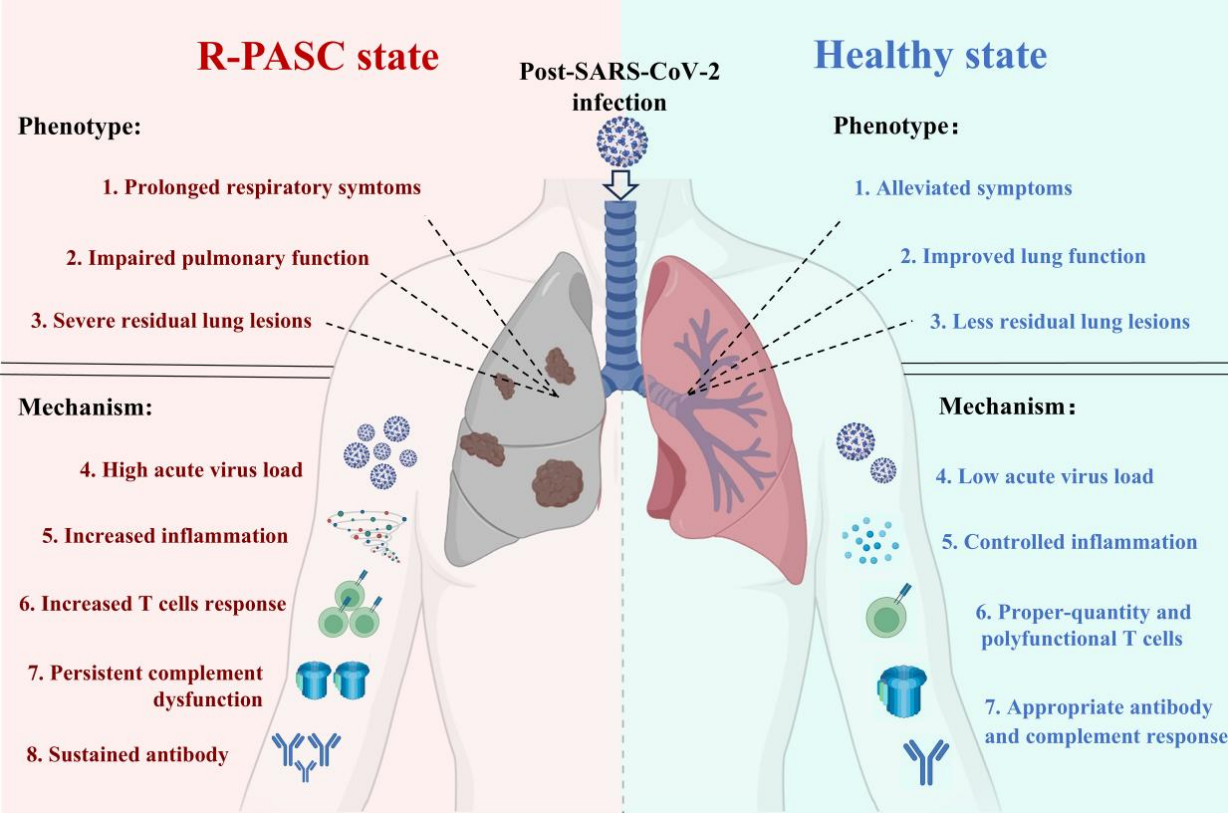
Schematic representation of clinical phenotypes and underlying pathogenic mechanisms in R-PASC state. The schematic diagram demonstrates the clinical phenotypic differences between patients with R-PASC and non-R-PASC healthy individuals, encompassing respiratory symptoms and parameters, and underlying virus-immune pathogenic mechanisms. R-PASC, respiratory system–specific postacute sequelae of COVID-19.

To date, multiple hypotheses regarding PASC pathogenesis have been proposed [4], with immune- and inflammation-related mechanisms being central among them [19]. However, these factors have largely been examined and discussed in isolation, and limited insights within the context of organ/system-specific PASC [13, 24, 25]. Herein, this study longitudinally evaluated the immunological, inflammatory, and clinical respiratory parameters in COVID-19 convalescent individuals with or without R-PASC. We comprehensively analyzed the respective disease impacts of the virus-specific T cells, antibodies, complement, and inflammatory molecules, thereby bridging the gap between systemic immune-inflammation dysregulation and lung-specific PASC pathophysiology (Figure 6).

T cells can form long-lasting immune memory following the clearance of the initial viral infection, providing protection to the host against reinfections [26, 27]. In the field of PASC, our observations also highlight their important protective role in respiratory PASC. Long-term SARS-CoV-2–specific T cells with polyfunctionality and appropriate responses magnitude correlated with improved respiratory function and alleviated inflammatory response, thus may mitigate R-PASC progression. This involves 2 key features of T cells: multifunctionality and quantity. During acute infection, multifunctional SARS-CoV-2–specific T-cell responses have been observed in mild/asymptomatic cases and are associated with early viral control [20, 28], whereas their presence is insufficient in severe cases and skews toward a cytotoxic/exhausted phenotype [29]. Meanwhile, the CMV-specific multifunctional CD4^+^ memory T cells are also found to mediate durable lung mucosa protection [30]. These observations reinforce the multifaceted protective roles of polyfunctional virus-specific T cells at various stages of viral infection, spanning from early viral clearance and prevention of reinfection to mitigation of postviral sequelae. Our findings also emphasize that while an appropriate quantity of virus-specific T cells is also crucial in R-PASC pathophysiology, a threshold-driven balance between protective immunity from tissue-resident memory T cells in lung and pathological infiltration must be established in the future.

On the other side, high and sustained T-cell responses with reduced naïve phenotype in R-PASC patients likely stems from residual viral antigen reservoirs, as evidenced by detectable SARS-CoV-2 RNA fragments in multiple organs months after infection [9]. Recent mass spectrometry studies confirm S1 protein persistence in CD16^+^ monocytes up to 15 months after infection in patients with PASC may perpetuate T-cell activation through prolonged antigen presentation [31]. Our findings of concurrent virus-specific T cell and antibody accumulation in multisystem PASC further support this paradigm. Notably, inflammation-driven bystander T-cell activation mediated by elevated inflammatory cytokines (eg, IL-6, IL-1β) may also contribute to polyclonal T-cell expansion in some patients with PASC [32, 33]. Furthermore, although this study did not observe any correlation between high virus-specific T-cell responses and disease indicators in patients with R-PASC, future investigations should carefully examine whether or to what extent these high virus-specific T-cell responses in PASC are associated with immunopathology [34].

The complement system is another crucial immune factor in the PASC pathophysiology. Imbalanced formation of the terminal complement complex has been reported in patients with Long COVID and persists for up to 6 months postacute infection [24]. Our data support and extend these findings, suggesting that the chronic complement hyperactivation in patients with R-PASC correlates with persistent pulmonary dysfunction and radiographic evidence of severe pulmonary lesions. Mechanistically, this pathological process involves coordinated dysregulation across all three complement pathways, particularly the terminal (sC5b-9), classical (C1q), and alternative (Factor B) pathways, mirroring recent reports of pathway-specific activation signatures in Long COVID cohorts [35]. Remarkably, MBL enrichment in individuals unaffected by PASC and its protective association with pulmonary recovery, identified in our study aligns with the emerging evidences of lectin pathway deficiency as a key susceptibility factor for Long COVID brain fog [36], as well as in chronic fatigue syndrome [37], a disease triggered by acute infection similar to PASC. The unresolved mechanistic basis of this observation, despite its ubiquity in postviral syndromes, underscores the urgent need for pathway-specific interrogation—a prerequisite for translating complement modulation into viable clinical interventions for PASC.

In summary, our study elucidates the dysregulated interplays between chronic immune activation, sustained inflammatory responses, and prolonged pulmonary injury in R-PASC pathogenesis (Figure 6). These findings also suggest the potential utility of virus-specific T cells and multiple complement as therapeutic targets for this condition. However, several limitations should be considered when interpreting these results. The nonmatched design of this prospective cohort precludes definitive causal inference between acute-phase injury and subsequent R-PASC and cannot fully rule out unmeasured confounding. Additionally, the relatively small sample size and the predominance of hospitalized patients limit the generalizability of our findings. Future studies incorporating larger outpatient cohorts, local immune profiling, and rigorous matching for acute disease severity are warranted to validate and extend our observations.

## METHODS

### Study Cohort

This prospective cohort study was conducted at The First People's Hospital of Yunnan Province during China's Omicron BA.5 outbreak. Our cohort primarily consisted of convalescent individuals who were hospitalized following their first SARS-CoV-2 infection and had previously received 2 to 3 doses of inactivated vaccines (Supplementary Table 3). Follow-up evaluations were performed at 4 and 7 M after initial infection. Each follow-up included: (1) physician-administered PASC symptom assessments via the PASC Symptom Assessment Form, (2) peripheral blood sample collection, and (3) clinical respiratory evaluations (pulmonary function tests and chest CT). Participation in follow-ups and respiratory assessments was voluntary.

PASC were defined according to the World Health Organization criteria (October 2021) [14]: (1) confirmed SARS-CoV-2 infection by quantitative reverse transcription polymerase chain reaction (PCR) or antigen testing; (2) symptoms emerging or worsening at least 3 months after COVID-19 onset, persisting for ≥2 months; and (3) exclusion of alternative diagnoses through clinical evaluation and medical history. Participants were categorized into organ/system-specific PASC groups based on the sustained symptoms aligned with corresponding physiological systems [38]: (1) respiratory system PASC (chronic cough, dyspnea); (2) cardiovascular system PASC (palpitation, chest pain); (3) neurological PASC (headache, cognitive impairment, fatigue, insomnia); (4) digestive system PASC (nausea, anorexia); (5) sensory system PASC (hyposmia, hypogeusia); and (6) musculoskeletal system PASC (myalgia, arthralgia). Notably, assignment to a specific organ-system PASC group (eg, respiratory PASC) was based on the presence of defining symptoms for each system, without excluding individuals with concurrent symptoms from other systems. This approach was chosen to reflect the clinical reality of frequent symptom overlap (Supplementary Table 4) in PASC while allowing us to focus on the pathophysiology of the targeted PASC system.

### Chest CT Protocol and Quantitative Scoring System

Serial Chest CT was performed for hospitalized COVID-19 convalescents in acute-phase, 4 and 7 M with commercial multidetector CT scanners (SOMATOM, Siemens, Germany). Two certified radiologists independently analyzed CT images using a semiquantitative scoring system (Chest CT Score) validated in prior studies [17, 18, 39, 40]. Each lung was divided into 5 lobes (right upper, middle, lower; left upper, lower), with individual lobe scores assigned as follows: 0 (no lesions), 1 (<5% involvement), 2 (5–25%), 3 (26–50%), 4 (51–75%), or 5 (>75%). Total scores ranged from 0 to 25, reflecting overall disease burden. Lesion morphology was characterized per Fleischner Society guidelines [41], including ground-glass opacity, consolidation, and fibrosis.

### Human Pulmonary Function Testing

Lung function assessments were conducted during acute SARS-CoV-2 infection and at 4 M after infection, with voluntary participation in follow-up testing. Measurements were performed in a standardized laboratory using a Masterscreen Jaeger spirometer (Germany), adhering to American Thoracic Society/European Respiratory Society guidelines [42]. Participants underwent 3 repeated tests at 5-minute intervals to minimize fatigue effects. Analyzed parameters included: FVC%, FEV1%, FVC/FEV1 ratio (ventilation), and DLCO%.

### Viral RNA Measurements

The nasopharyngeal swab samples collected during the acute phase of SARS-CoV-2 infection were tested for viral RNA using a commercial kit (Shengxiong Biotechnology Co., Ltd., Changsha, China) via quantitative reverse transcription PCR. This kit targets the ORF1ab and N genes and has been approved by the National Medical Products Administration of China. Samples were included for analysis within 20 days postsymptom onset.

### SARS-CoV-2 Conventional Virus Neutralization Test

To assess viral-neutralizing capacity, plasma specimens underwent analysis via a cytopathic response assay conducted in an certified biosafety level 3 facility, as described previously [43]. In brief, serially diluted plasma samples were incubated with viral suspensions (SARS-CoV-2 Omicron BA.5 or XBB 1.9 subvariants) before inoculation onto VERO E6 cells. After a 4-day culture, neutralizing antibody titers were quantified through automated cytopathic effect analysis (Celigo Imaging System, Nexcelom Bioscience), with geometric mean values calculated from duplicate measurements relative to virus and cell controls.

### iFlash-SARS-CoV-2 IgG Assay

Quantification of SARS-CoV-2–specific IgG antibodies targeting both spike (S) and nucleocapsid (N) proteins (anti-S + N IgG) was conducted using a chemiluminescent immunoassay platform (iFlash 3000, Yhlo Biotech, Shenzhen, China) in accordance with the manufacturer's instructions, as described previously [44].

### Cytometric Bead Array

Plasma concentrations of inflammatory cytokines and chemokines (IL-6, IL-8, IP-10, MIP-1α, MCP-1, IFNγ, TNF) were quantified using the Cytometric Bead Array Human Soluble Protein Flex Set System (BD Biosciences, USA) following manufacturer protocols. Briefly, following serum centrifugation to remove debris, supernatants were analyzed alongside serially diluted cytokine standards. Antibody-conjugated magnetic beads were incubated with samples and detection reagents, followed by flow cytometric analysis (BD Fortessa). Absolute concentrations were determined via FCAP Array software using manufacturer-defined standard curves.

### PBMCs ex Vivo Stimulation and Flow Cytometry

PBMCs stimulation protocols employing SARS-CoV-2 peptide pool mixtures were implemented following established methodologies [15, 43]. In brief, PBMCs were activated ex vivo with SARS-CoV-2 peptide pools (250 nM/peptide), recombinant IL-2 (20 IU/mL; PeproTech, USA), and protein transport inhibitor GolgiPlug (1 μM; BD Biosciences, USA) for 16 hours.

Following overnight culture, cells were stained with Live/Dead-FVS440 (BD, 1:1000, #566332) for viability assessment, followed by surface marker labeling using CD3-BUV395 (BD, 1:200, #564001, SK7), CD4-APC-H7 (BD, 1:150, #560158, RPA-T4), CD8-PerCP-Cy5.5 (BioLegend, 1:200, #344710, SK1), CCR7-APC (BioLegend, 1:150, #353214, G043H7), and CD45RA-FITC (BioLegend, 1:200, #304106, HI100). After fixation/permeabilization (BD Cytofix/Cytoperm), intracellular cytokines were detected with IFN-γ-BV421 (BioLegend, 1:200, #506538, B27) and TNF-α-PE (BD, 1:200, #559321, MAb11). Washed cells were analyzed on a BD Fortessa X-20 flow cytometer, with data processed using FlowJo v10.8.

### Complement Factor Measurements

The levels of complement system components (Sc5b-9, C3a, C5a, C1q, MBL, and Factor B) were quantitatively analyzed in cell-free plasma samples using commercially available enzyme-linked immunosorbent assay kits (Jiangsu Meimian Industrial Co., Ltd., China). Following standardized enzyme-linked immunosorbent assay protocols, diluted plasma samples and reference standards were loaded into designated wells, followed by sequential incubation with horseradish peroxidase–conjugated detection antibodies. After chromogenic reactions initiated by substrate addition, optical density at 450 nm was measured using a Multiskan GO microplate spectrophotometer (Thermo Fisher Scientific, USA).

### Statistical Analyses

All data analyses were conducted using GraphPad Prism (v9.5.1) and SPSS (v26), with flow cytometry data processed in FlowJo. Nonnormally distributed data were analyzed using 2-tailed Mann-Whitney *U* tests, with results presented as median and interquartile range. Normally distributed data were compared via Student *t*-test and expressed as mean ± standard error of the mean. Antibody titers were reported as geometric mean titers with 95% confidence intervals. Paired nonparametric comparisons used the Wilcoxon signed-rank test. CT pulmonary feature changes between timepoints were assessed using the chi-squared test. Correlations were assessed using Spearman's rank correlation coefficient and linear regression analysis. Statistical significance thresholds were defined as *P* < .05 (*), *P* < .01 (**), and *P* < .001 (***).

## Supplementary Material

ofag050_Supplementary_Data

## Supplementary Data

Supplementary materials are available at *Open Forum Infectious Diseases* online. Consisting of data provided by the authors to benefit the reader, the posted materials are not copyedited and are the sole responsibility of the authors, so questions or comments should be addressed to the corresponding author.
