# Supplementary material for: Extensive Immunological and Inflammatory Perturbation Underpins the Respiratory Sequelae of Postacute COVID-19
Source: Open Forum Infect Dis. 2026 Feb 5;13(2):ofag050. doi: 10.1093/ofid/ofag050 (PMC12937584; doi:10.1093/ofid/ofag050)
Supplement: ofag050_Supplementary_Data [file ofag050_supplementary_data.zip › Supplementary Data - PASC Symptom Assessment Form.docx]

**PASC Symptom Assessment Form**

Patient ID: _________ Gender: _________ Age: _________

Assessing Physician: _________ Date: _________

1. Date of initial symptom onset of Omicron infection: _________
2. Date of first positive nucleic acid/antigen test of Omicron infection: _________
3. Have you been infected with SARS-CoV-2 between 2020 to 2023? □ No □ Yes
4. Do you currently cough?

4.1 Is it newly occurring after Omicron infection? □ No □ Yes

4.2 Duration in days: _______________

1. Do you currently feel dyspnea?

5.1 Is it newly occurring after Omicron infection? □ No □ Yes

5.2 Duration in days: _______________

1. Do you currently feel palpitation?

6.1 Is it newly occurring after Omicron infection? □ No □ Yes

6.2 Duration in days：_______________

1. Do you currently feel chest pain?

7.1 Is it newly occurring after Omicron infection? □ No □ Yes

7.2 Duration in days：_______________

1. Do you currently feel fatigue?

8.1 Is it newly occurring after Omicron infection? □ No □ Yes

8.2 Duration in days：_______________

1. Do you currently feel nausea or anorexia?

9.1 Is it newly occurring after Omicron infection? □ No □ Yes

9.2 Duration in days：_______________

1. Do you currently feel headache?

10.1 Is it newly occurring after Omicron infection? □ No □ Yes

10.2 Duration in days：_______________

1. Do you currently feel loss of smell and taste?

11.1 Is it newly occurring after Omicron infection? □ No □ Yes

11.2 Duration in days：_______________

1. Do you currently feel decreased reaction time and impaired memory?

12.1 Is it newly occurring after Omicron infection? □ No □ Yes

12.2 Duration in days：_______________

1. Do you currently feel insomnia and early awakening?

13.1 Is it newly occurring after Omicron infection? □ No □ Yes

13.2 Duration in days：_______________

1. Do you currently feel anxiety and emotional instability?

14.1 Is it newly occurring after Omicron infection? □ No □ Yes

14.2 Duration in days：_______________

1. Do you currently feel muscle pain?

15.1 Is it newly occurring after Omicron infection? □ No □ Yes

15.2 Duration in days：_______________

1. Do you currently feel joint pain?

16.1 Is it newly occurring after Omicron infection? □ No □ Yes

16.2 Duration in days：_______________
