# Supplementary figures and images for: Extensive Immunological and Inflammatory Perturbation Underpins the Respiratory Sequelae of Postacute COVID-19
Source: Open Forum Infect Dis. 2026 Feb 5;13(2):ofag050. doi: 10.1093/ofid/ofag050 (PMC12937584; doi:10.1093/ofid/ofag050)

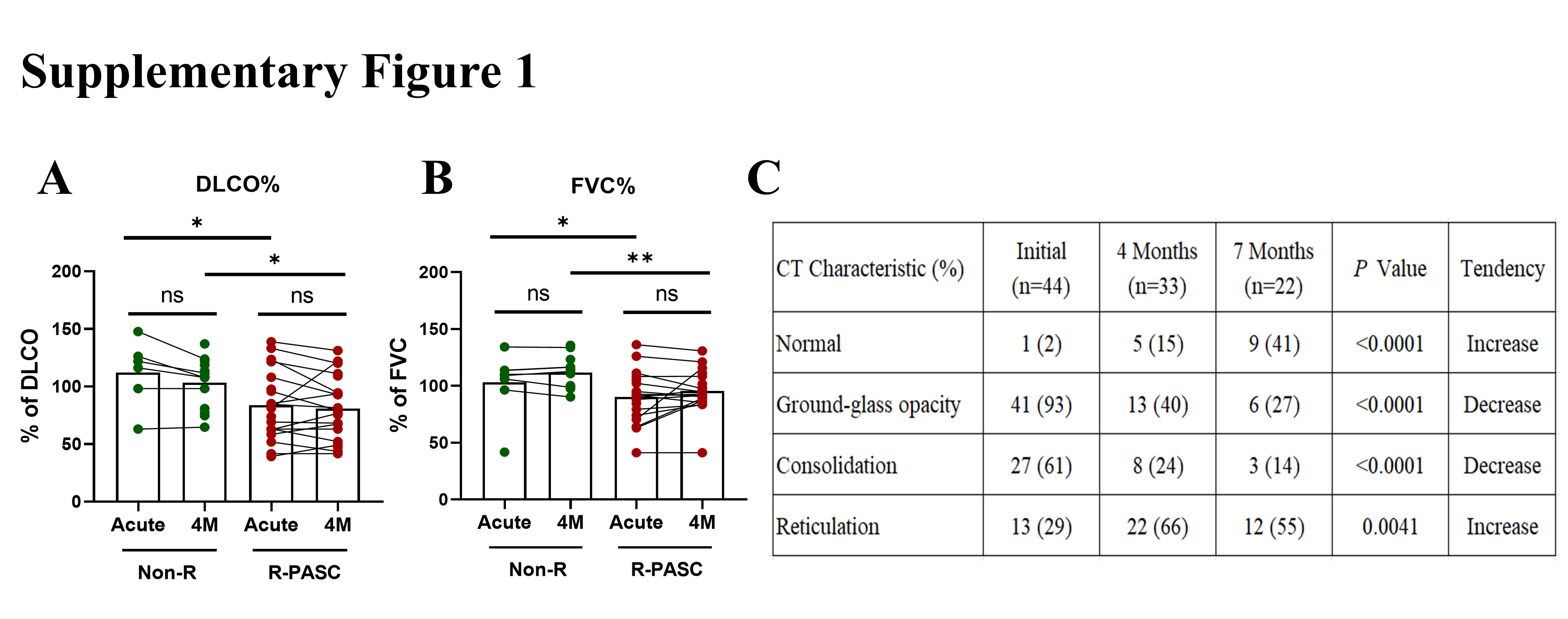

Supplement: ofag050_Supplementary_Data [file ofag050_supplementary_data.zip › Supplementary Figure 1.TIF]

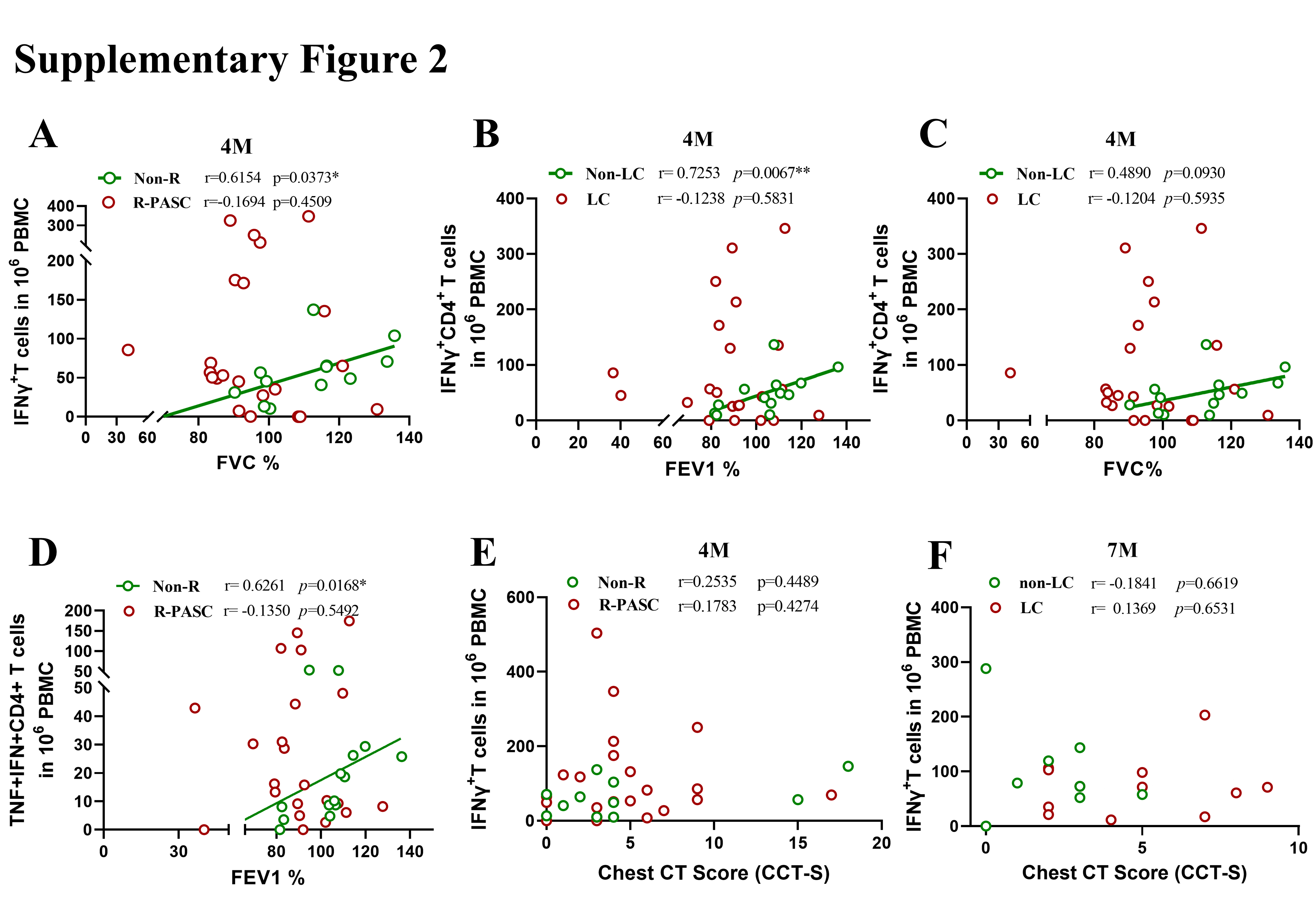

Supplement: ofag050_Supplementary_Data [file ofag050_supplementary_data.zip › Supplementary Figure 2.TIF]

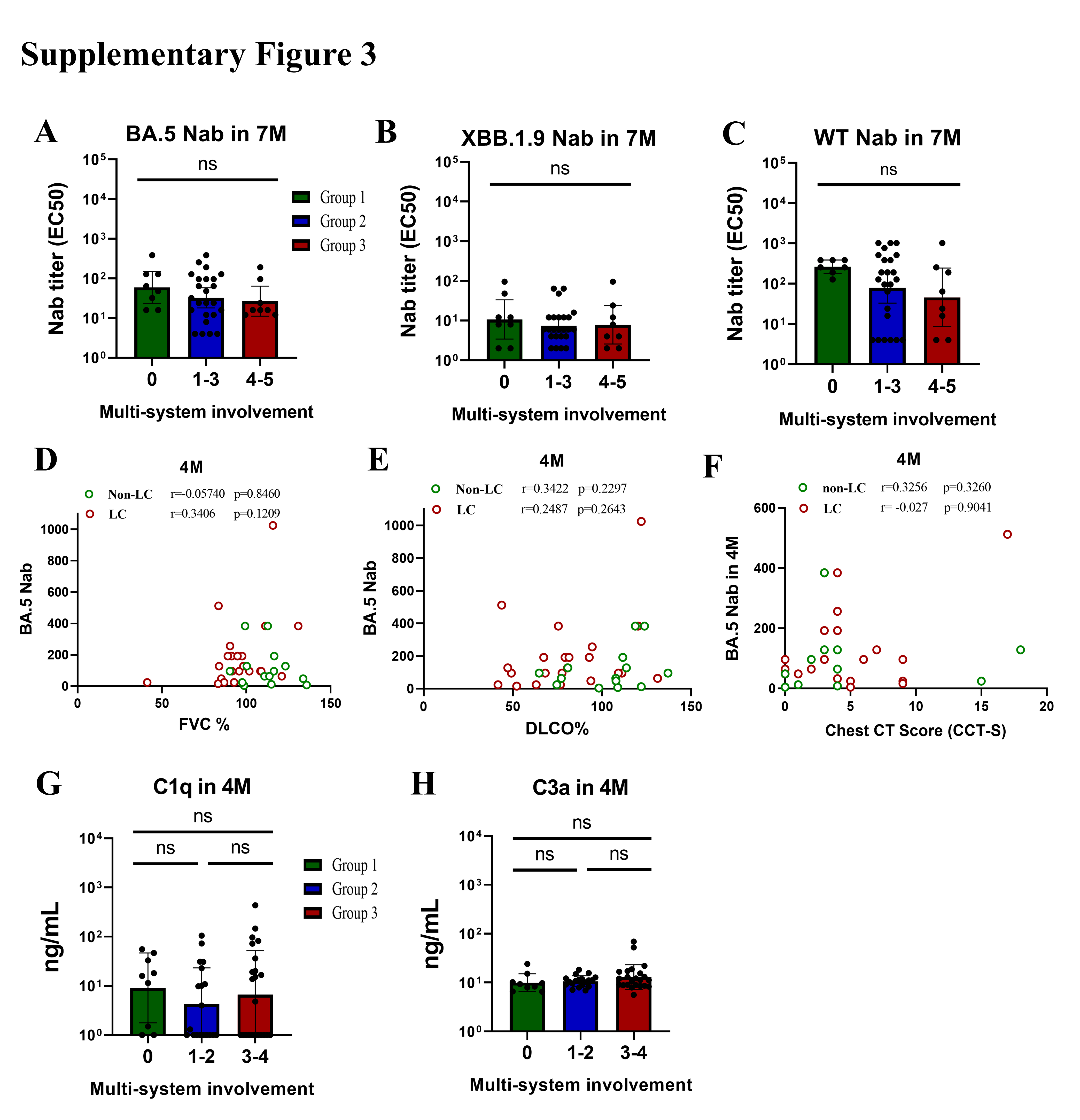

Supplement: ofag050_Supplementary_Data [file ofag050_supplementary_data.zip › Supplementary Figure 3.TIF]
